# Investigating Hydroclimatic Variables Trends on the Natural Lakes of Western Greece Using Earth Observation Data

**DOI:** 10.3390/s23042056

**Published:** 2023-02-11

**Authors:** Nikolaos Gourgouletis, Evangelos Baltas

**Affiliations:** Department of Water Resources & Environmental Engineering, School of Civil Engineering, National Technical University of Athens, Str. Iroon Politexniou 9, 157 80 Zografou, Greece

**Keywords:** climate change, lakes, remote sensing, water area, trend analysis, ERA5, Landsat

## Abstract

Expected global climate change is allegedly becoming more intense, and the impacts on water resources are being tracked in various hydroclimatic regimes. The present research investigates a hydrologically important area of Greece, where four natural lakes are concentrated. It aims to quantify any potential long-term trends in lake water area, precipitation, and temperature timeseries. Water area timeseries spanning four decades are estimated by the mNDWI from Landsat satellite imagery and used as an index of each lake’s water storage. Precipitation and temperature measurements are obtained from the open access datasets *Hydroscope* and ERA5-Land, respectively. All of the timeseries were tested seasonally and annually with the Pettitt and Mann–Kendal tests for statistically significant breakpoints and trends detection. No timeseries analysis resulted in a statistically significant (at 0.05 or 0.1 levels) annual or seasonal trend. The hydroclimatic regime over the past forty years in western Greece is found to have been relatively stable. Land use was also assessed to have been relatively unchanging, converging to the overall stability of the local water regime. However, the findings of this research should not be interpreted as a reassurance against climate change, but as a call to further research for the detailed regional and local assessment of climate change and hydroclimatic variability with acknowledged statistical approaches.

## 1. Introduction

Natural lakes and manmade reservoirs play a key role in the Earth’s water cycle, satisfying the water demands of human activities along with important ecosystem services [1,2,3,4]. They also are important indexes of climate conditions, trends, and abrupt changes, with deviations of their physical, chemical, and biological features [1,5]. Lakes and reservoirs regulate river flows, mitigate floods, provide groundwater recharge flows, improve and stabilize water quality, as well as frequently serving as the primary water supply source for human use [3,6]. Additionally, lakes and reservoirs hold a significant share in the global carbon cycle and contribute to methane emissions [1,3,7]

Climate change is projected to augment the pressures and intensify the impacts on water resources, especially in the mid latitudes—Mediterranean region [8,9,10,11]. Thus, alterations of the water cycle are expected, highlighting its importance on providing ecosystem services and regulating extreme flows [8,9]. Furthermore, land cover/use changes subject to economic growth, increased livestock, increased need of pasture and agricultural production, as well as increased water demands, constitute another key pressure on water resources [2,12,13,14,15].

Greece is a typical Mediterranean country, where in recent decades, land resources are documented to be overexploited, while noteworthy shares of land have been transformed into artificial surfaces, intensifying the pressures on water and land resources [16]. Moreover, after the mid-20th century, an increasing number of droughts is being recorded in Greece, and relevant studies project an intensification of the drought phenomena, along with incremental water stress [17,18,19]. It is worth noting a drought occurrence that extended to the whole country in 1990, which is considered the most severe of the 20th century [20]. By the time of the drought’s peak, in December of 1990, the water reserves of the country’s capital, Athens, were expected to only last for a few weeks more [21]. 

Considering the issues mentioned above, the quantitative pressures exerted on water resources are expected to increase in the near future. The latter will also be required to contribute to an increasing demand of the human environment, as local regulators of climate change and as pillars sustaining ecosystems. A major role of any toolkit of sustainable water resource management in defence of water security, is stated to be their monitoring [22,23,24]. Water resource monitoring is feasible with in-situ observations, hydrological modelling, and remote sensing techniques [25]. From the 1990s, remote sensing approaches towards the monitoring of inland waters have been receiving increasing consideration from researchers and governance bodies. The most important aspects of the lakes’ and reservoirs’ water cycles, their water levels, water areas, and water storages have been attracting the highest research interest.

The Normalized Difference Water Index (NDWI) was introduced in 1996, and constitutes the most widely adopted index for delineating water features from satellite imagery [26,27,28,29,30]. However, the modified NDWI (mNDWI) has gained significant approval, and is considered to be more accurate in the distinction of water bodies [31,32,33,34,35,36]. The modification consists of replacing the Near Infrared Band spectrum used in the NDWI, with a higher bandwidth, middle infrared band. Thus, the mNDWI has a higher accuracy and less noise, when delineating open water features, compared to the original NDWI [37]. 

Since the first launch of the Landsat-1 satellite in 1972, environmental earth observation entered a new phase. Optical satellite imagery provides an almost 50 year-long record of important measurements over water bodies. Although altimetry missions became adequate sources of water level information a few decades ago, water area estimations are feasible for a much longer timeseries, constituting a significant source of information for long-term hydroclimatic analysis [14]. 

Accordingly, this research aims to assess the long-term trends in hydroclimatic variables in western Greece, where several natural lakes are located. Despite the large amounts of available consistent remote sensing data concerning quantitative indices of the water cycle, only a couple of studies have focused on these aspects of the lake water cycle, e.g., water area, water level, or water storage [30,38]. However, there have been several applications of remote sensing techniques on the qualitative characteristics of inland water bodies [39,40,41,42,43,44,45,46]. Hence, this research aims to target the long-term climatic trends of the natural lakes of western Greece, Trichonis, Lysimacheia, Ozeros, and Amvrakia, using remote sensing water area estimations as the main water content indicator. The abovementioned choice, of the water area as an indirect index of water storage, is considered justified due to the converging conclusions of various studies towards the high correlation between water area and water storage timeseries [2,24,25,30,47,48,49,50]. Moreover, this research aims to analyse the simultaneous trends of importance to the water cycle climate variables, such as precipitation and temperature, as well as human interference in the land use pattern of each lake basin.

## 2. Materials and Methods

### 2.1. Study Area

**Figure 1:**
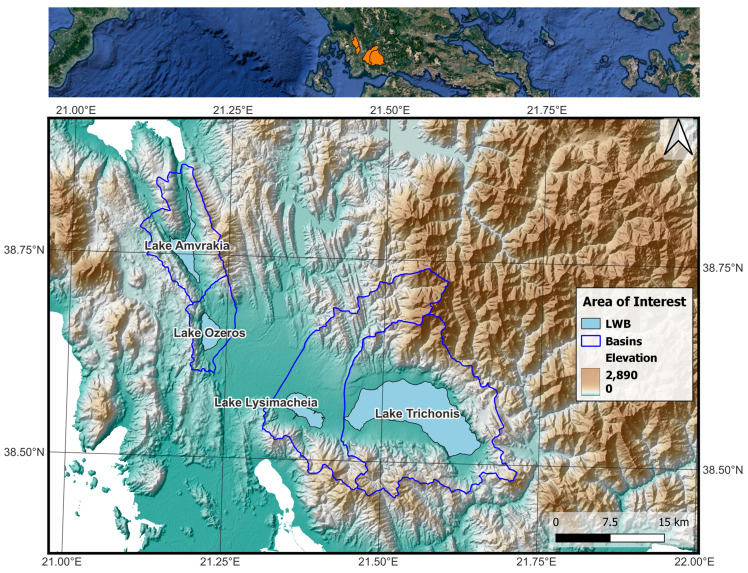
**Upper***:* Basins of lakes Trichonis, Lysimacheia, Ozeros, and Amvrakia, and their locations in Greece. **Lower***:* Detailed view of the above lakes (LWB) and basins in SW part of western Greece with a digital elevation model background.

The four natural lakes, Trichonis, Lysimacheia, Ozeros, and Amvrakia are located in western Greece, a rich hydrologic part of Greece, and are presented along with their corresponding basins in Figure 1. All four lakes belong to the River Basin District of western Greece, which is characterised by the second highest average precipitation in the country. Specifically, low elevation regions receive 800–1000 mm annual precipitation, while mountainous regions receive 1400–1800 mm of annual precipitation. The dominant land cover/land uses are forests (> 45%) and pastures (> 30%), followed by crop cultivation and roads [51].

Lake Trichonis is the largest lake in Greece, with a nominal water area of 96.51 km^2^ and water perimeter of around 53.5 km. It is a protected area of aquatic species of economic importance under the Directive 2006/44/EC, due to its significant production of smelt. Its annual inflows are 254 hm^3^ from its hydrologic basin, as well as 131.5 hm^3^ coming from a diversion channel from the hydroelectric reservoir Stratos II. About 329.6 hm^3^ on average are diverted from lake Trichonis to lake Lysimacheia, while the direct water abstractions from lake Trichonis account for 12.96 hm^3^. Under the 1st Revision of the River Basin Management Plan of Western Greece, lake Trichonis is considered to have a good ecological and chemical status. Lake Trichonis is considered to have an average depth of 30.5 m and a maximum depth of 57.0 m, about 40 m below sea level [52,53]. Moreover, lake Trichonis is indexed as a NATURA 2000 protection area, as an area of high potential research value [52].

Lake Lysimacheia has a nominal water area of 13.04 km^2^ and water perimeter of around 22.9 km. Its annual inflows are 123 hm^3^ from its hydrologic basin, as well as 329.6 hm^3^ coming from a diversion channel from lake Trichonis. About 264.7 hm^3^ on average are diverted from lake Lysimacheia to the nearby Acheloos river basin, while the direct water abstractions from lake Lysimacheia account for 6.3 hm^3^. Under the 1st Revision of the River Basin Management Plan of Western Greece, lake Lysimacheia is considered to have a moderate ecological and good chemical status.

Lake Ozeros has a nominal water area of 9.39 km^2^ and water perimeter of around 13.6 km. Its annual inflows are 24.3 hm^3^ from its hydrologic basin. Lake Ozeros is not considered to receive any water abstractions. Under the 1st Revision of the River Basin Management Plan of Western Greece, lake Ozeros is considered to have a moderate ecological and good chemical status.

Lake Amvrakia has a nominal water area of 14.53 km^2^ and water perimeter of around 34.35 km. Its annual inflows are 84.0 hm^3^ from its hydrologic basin. Lake Amvrakia is not considered to receive any known water abstractions. However, there is an evident decrease of its water content in its north compartment, which has not been attributed to a specific cause. Under the 1st Revision of the River Basin Management Plan of Western Greece, lake Ozeros is considered to have a good ecological and chemical status [51,54,55].

### 2.2. Data Used

#### 2.2.1. Landsat Family IMAGERY

Satellite images from the Landsat missions 4, 5, 7, 8, and 9, covering the four lakes’ basins, were collected from the United States Geological Survey (USGS) online portal (https://earthexplorer.usgs.gov/, accessed on 1 December 2022). Landsat 4, launched in 1982, was the first environmental remote sensing satellite to provide band products at the resolution of 30 m, which is sufficient to capture the examined lakes. Thus, the selected dataset covers the temporal period between 1984–2022, with a spatial resolution of 30 m, for every Landsat mission mentioned. The selection of 1984 as a start year is based on satellite products’ availability and temporal consistency. Moreover, the selected dataset had cloud covered images removed, resulting in 375 satellite images throughout the examined years, which leaves approximately 10 images per year. Regarding Landsat 7’s SLC failure, the present research followed the gap-filling methodology prescribed by the USGS [56]. Regarding the rest of the Landsat missions, 4, 5, 8, and 9, it should be noted that their products selected are Level-2, therefore already preprocessed and ready to use for the scope of the present research.

#### 2.2.2. In-Situ Precipitation Measurements

In-situ daily precipitation measurements for the four lakes’ basins were collected from the “Hydroscope” project’s online portal (http://www.hydroscope.gr/, accessed on 1 December 2022). Fourteen hydrometeorological stations were selected within the basins of the four lakes and their precipitation data were downloaded from the portal. The stations’ locations are Analipsi, Gavalou, Thermo, Kallithea, Lepenou, Poros Riganiou, Stamna, Ag. Vlasios, Achira, Monastirakion, Sargiada, Stanos, Lesinio, and Trikorfo. Their altitude varies from 2 m.a.s.l. to 852 m.a.s.l., and they are all operated by the Hellenic Ministry of Environment and Energy. The available datasets cover almost the entirety of the water area timeseries, containing the years from 1984 until 2016–2019. 

#### 2.2.3. ERA5-Land Monthly Temperature Data

ERA5-Land reanalysis data for the four lakes’ basins were collected from the EU Copernicus Climate Change Service online portal (https://cds.climate.copernicus.eu, accessed on 1 December 2022). The ERA5-Land reanalysis dataset provides 2 m above ground temperature data at a maximum temporal resolution of one hour, and a spatial resolution of 0.1 degree. For the purpose of the present research, the downloaded data consisted of the monthly average temperatures, containing the years from 1984 until 2022, fully covering the water area timeseries. ERA5-Land reanalysis products are considered ideal in cases of scarce in-situ data, as in the case of western Greece where there is a lack of open access temperature data. Moreover, ERA5-Land data have a growing usage in hydrology and remote sensing applications [57,58], thus they are considered suitable for the purposes of the present research. 

#### 2.2.4. CORINE Land Cover (CLC)

Land cover data for the four lakes’ basins were collected from the EU Copernicus Land Monitoring Service online portal (https://land.copernicus.eu/, accessed on 1 December 2022). The CLC dataset is characterized by a minimum mapping unit of 25 ha and a minimum mapping width of 100 m [59]. Two datasets covering western Greece were downloaded from the abovementioned portal, one covering the time period around year 1990 and the other covering the time period around year 2018. 

### 2.3. Methodology

#### 2.3.1. Water Area Extraction

For the calculation of the mNDWI, the Green and SWIR bands were used and the water and non-water pixels were distinguished following Equation (1) [32,33,34,35]. The mNDWI is characterized by a spatial resolution of 30 m, deriving from the resolution of the Landsat bands used. For each Landsat mission used, the different bands’ names representing Green and SWIR are presented in Table 1.

(1) $$\mathrm{mNDWI}=\frac{\mathrm{Green}-\mathrm{SWIR}}{\mathrm{Green}+\mathrm{SWIR}}$$

Moreover, the nominal water–non water threshold value of 0, was substituted from an optimum threshold following the minimum thresholding method, initially proposed by Prewitt and Mendelsohn [60]. The selected approach has been proven to be more accurate than other thresholding approaches [29], and has also been tested by the authors for the calculation of NDWI in Yliki reservoir [30]. Each date’s initial mNDWI’s histogram is smoothed until it shows only two local maxima, and the optimum threshold lies in the minimum value between them. The extracted wet perimeter is converted to a lake water polygon and the area is calculated for each date. 

#### 2.3.2. Statistical Point of Change and Trend Analysis

A widely accepted approach to detect statistically significant trends and points of abrupt change in hydrological variables is the combination of the Mann–Kendall [61,62] and Pettitt [63] tests, respectively. The abovementioned statistical tests have been applied extensively to hydroclimatic variables, including discharge timeseries [64,65,66], lake water level timeseries [14], as well as climatic variables including precipitation and temperature [14,64,65,66,67].

The statistic value U_t,N_ of a timeseries x_t_ (t = 1, 2, 3, …, N) is calculated as shown in Equation (2).

(2) $$U_{t,N}=U_{t-1,N}+V_{t,N}\;;\;t=2,\;3,\;\ldots ,\;N$$

where N is the timeseries size and V_t,N_ as described in Equation (3).

(3) $$V_{t,N}=\overset{N}{\underset{i=1}{\sum }}\mathrm{sgn}\left(x_{i}-x_{t}\right)$$

The location of the breakpoint K_N_, which represents the point of abrupt change, is defined by Equation (4) [63].

(4) $$K_{N}=\mathrm{Max}\;\left|U_{t,N}\right|\;;\;t=1,\;2,\;3,\;\ldots ,\;N\;$$

while the statistical significance *p* of K_N_ is approximated by Equation (5).

(5) $$p≅2\times \exp\left[\frac{-6\times K_{N}^{2}}{N^{3}+N^{2}}\right]$$

The null hypothesis “H_o_: no change point exists” is rejected at low *p*-values, and thus there is a significant point of abrupt change that separates the timeseries into two parts, pre and post change.

The Mann–Kendall test statistics S_MK_ are defined in Equation (6).

(6) $$S_{\mathrm{MK}}=\overset{N}{\underset{i}{\sum }}\overset{N}{\underset{j=i+1}{\sum }}\mathrm{sgn}\left(x_{j}-x_{i}\right)$$

For N ≥ 8, S_MK_ follows an approximately normal distribution and its variance, $\sigma_{\mathrm{MK}}^{2}$, is calculated as in Equation (7).

(7) $$\sigma_{\mathrm{MK}}^{2}=\frac{N\times \left(N-1\right)\times \left(2\times N+5\right)}{18}$$

Finally, the Mann–Kendall main test statistic Z_MK_ is defined in Equation (8).

(8) $$Z_{\mathrm{MK}}=\{\begin{array}{c} \frac{S_{\mathrm{MK}}-1}{\sigma_{\mathrm{MK}}^{2}},{\;\;\;\;S}_{\mathrm{MK}}>0\;\\ 0,\;{\;\;\;\;\;\;S}_{\mathrm{MK}}=0\\ \frac{S_{\mathrm{MK}}+1}{\sigma_{\mathrm{MK}}^{2}},{\;\;\;\;S}_{\mathrm{MK}}<0 \end{array}$$

The null hypothesis “H_o_: no trend detected” can be rejected at 5% significance level, *p*-value < 0.05, when |Z_MK_| > 1.96. If the above is true, then a positive value of Z_MK_ indicates a positive (incremental) trend, while a negative value of Z_MK_ indicates a negative trend.

The Mann–Kendall test, as defined in Equations (6)–(8), does not account for seasonality in the examined data. Hence, since many hydrologic and climatic variables display seasonality, an alteration of the MK test is applied. The alteration, proposed by Hirsch and Slack [68], is defined again by Equations (6)–(8), by substituting N (total timeseries sample size) with n_k_, which stands for the measurements size in season k. For the purposes of the present research, two seasons are assessed, the dry hydrological period (May to October) and the wet hydrological period (November to April).

## 3. Results

### 3.1. Water Area Timeseries and Trends

#### 3.1.1. Water Area Timeseries

The extraction of the lakes’ water areas, spanning from 1984 to mid-2022, resulted in 377, 376, 379, and 369 water area measurements for lakes Trichonis, Lysimacheia, Ozeros, and Amvrakia, respectively.

**Figure 2:**
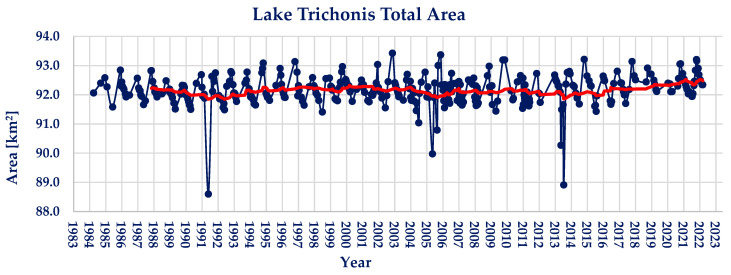
Landsat family derived lake Trichonis water area timeseries (red line; 2-year moving average).

Lake Trichonis’ water area measurements are presented in Figure 2. The maximum area observed is 93.43 km^2^, whilst the minimum area observed is 88.60 km^2^. The average water area of lake Trichonis during the observed period of 39 years is found to be 92.16 km^2^. During the period examined, lake Trichonis shows small percentages of water area changes, hence the maximum water extent is 1.38% larger than the average, and the minimum is 3.86% smaller. 

Regarding the Pettitt and Mann–Kendall tests conducted on the timeseries, there does not exist a statistically significant trend, although a breakpoint is identified in 2014. The calculated value of *p* is equal to 0.00 and Z_MK_ = −0.33. The absence of a statistically significant trend can be illustrated in the moving average trendline (red colored) shown in Figure 2.

**Figure 3:**
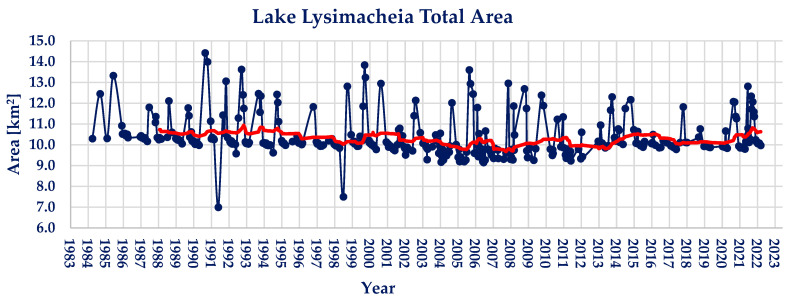
Landsat family derived lake Lysimacheia water area timeseries (red line; 2-year moving average).

Lake Lysimacheia’s water area measurements are presented in Figure 3. The maximum area observed is 14.42 km^2^, whilst the minimum area observed is 7.00 km^2^. The average water area of lake Lysimacheia during the observed period of 39 years is found to be 10.33 km^2^. During the period examined, lake Lysimacheia shows significant water area changes, with the maximum water extent being 39.61% larger than the average, and the minimum being 32.37% smaller.

Regarding the Pettitt and Mann–Kendall tests conducted on the timeseries, there does not exist a statistically significant point of change or trend, hence the calculated values of *p* ≈ 1 and Z_MK_ = −5.07. The absence of a statistically significant trend is illustrated in the moving average trendline (red colored) in Figure 3.

**Figure 4:**
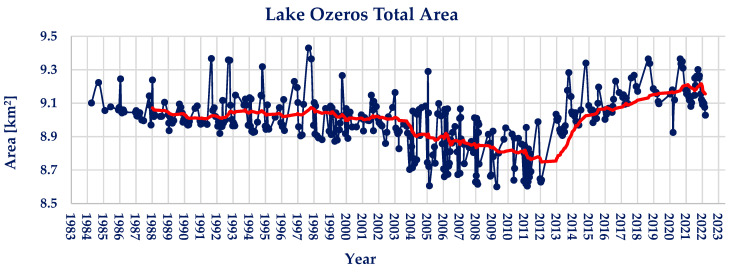
Landsat family derived lake Ozeros water area timeseries (red line; 2-year moving average).

Lake Ozeros’ water area measurements are presented in Figure 4. The maximum area observed is 9.43 km^2^, whilst the minimum area observed is 8.60 km^2^. The average water area of lake Ozeros during the observed period of 39 years is found to be 9.00 km^2^. During the period examined, lake Ozeros shows relatively small water area changes, with the maximum water extent being 4.81% larger than the average, and the minimum being 4.43% smaller.

Regarding the Pettitt and Mann–Kendall tests conducted on the timeseries, there does not exist a statistically significant point of change or trend, hence the calculated values of *p* ≈ 1 and Z_MK_ = +0.36. The absence of a statistically significant trend can be seen in the moving average trendline (red colored) shown in Figure 4.

**Figure 5:**
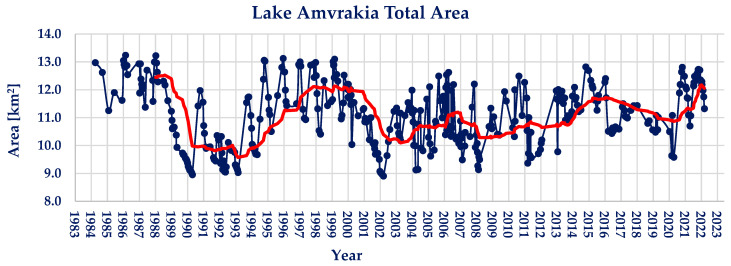
Landsat family derived lake Amvrakia water area timeseries (red line; 2-year moving average).

Lake Amvrakia’s water area measurements are presented in Figure 5. The maximum area observed is 13.24 km^2^, whilst the minimum area observed is 8.91 km^2^. The average water area of lake Amvrakia during the observed period of 39 years is found to be 11.10 km^2^. During the period examined, lake Amvrakia shows moderate water area changes, with the maximum water extent being 19.34% larger than the average, and the minimum being 19.71% smaller.

Regarding the Pettitt and Mann–Kendall tests conducted on the timeseries, there does not exist a statistically significant point of change or trend, hence the calculated values of *p* ≈ 1 and Z_MK_ = +1.87. The absence of a statistically significant trend can be seen in the moving average trendline (red colored) shown in Figure 5.

#### 3.1.2. Annual Water Area Trend Analysis

**Figure 6:**
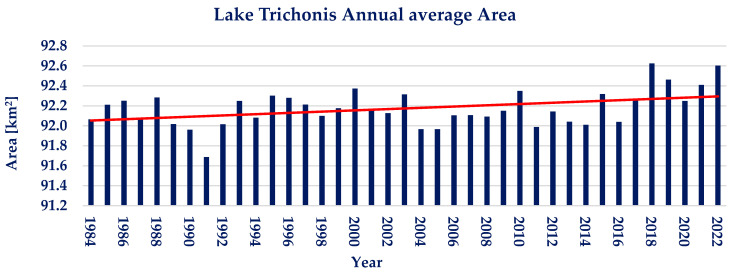
Annual water area average and linear trendline of lake Trichonis (red line; linear trendline).

The annual average of water area for lake Trichonis is presented in Figure 6. It is calculated that no significant trend is detected, despite a graphically observed slight positive trend. Lake Trichonis shows insignificant interannual water area variations during the examined 39 years, with the annual water area varying between −0.53% and +0.49% from the annual water area average of 92.17 km^2^. The Pettitt test conducted on the available annual timeseries does not show a statistically significant point of change. Moreover, the Mann–Kendall test resulted in *p* ≈ 1 and Ζ_ΜΚ_ = 1.84, depicting a nonsignificant positive trend.

**Figure 7:**
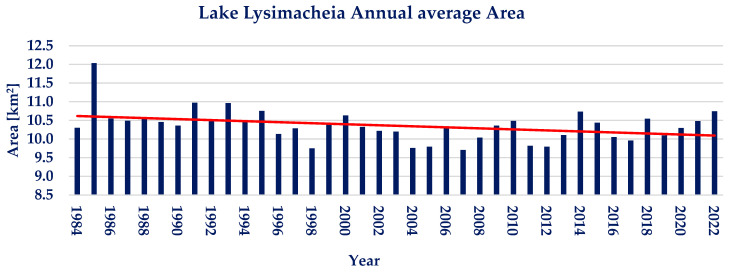
Annual water area average and linear trendline of lake Lysimacheia (red line; linear trendline).

The annual average of the water area for lake Lysimacheia is presented in Figure 7. It is calculated that no significant trend is observed, despite a graphically observed negative trend. Lake Lysimacheia shows small interannual water area variations during the examined 39 years, with the annual water area varying between −6.27% and +13.94% from the annual water area average of 10.35 km^2^. The Pettitt test conducted on the available annual timeseries does not show a statistically significant point of change. Moreover, the Mann–Kendall test resulted in *p* ≈ 1 and Ζ_ΜΚ_ = −2.06, depicting a nonsignificant negative trend.

**Figure 8:**
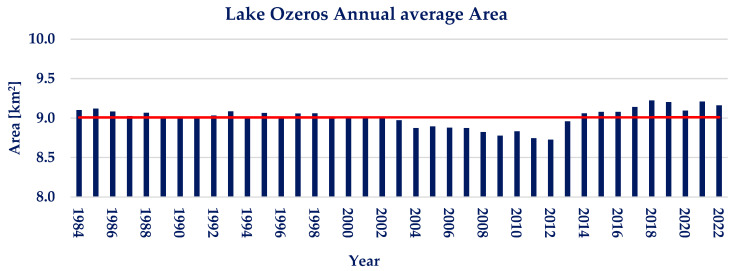
Annual water area average and linear trendline of lake Ozeros (red line; linear trendline).

The annual average of water area for lake Ozeros is presented in Figure 8. It is clearly depicted that no significant trend is observed. Lake Ozeros shows small interannual water area variations during the examined 39 years, with the annual water area varying between −3.14% and +2.31% from the annual water area average of 9.0 km^2^. The Pettitt test conducted on the available annual timeseries does not show a statistically significant point of change. Moreover, the Mann–Kendall test resulted in *p* ≈ 1 and Ζ_ΜΚ_ = −0.60, showing a nonsignificant negative trend.

**Figure 9:**
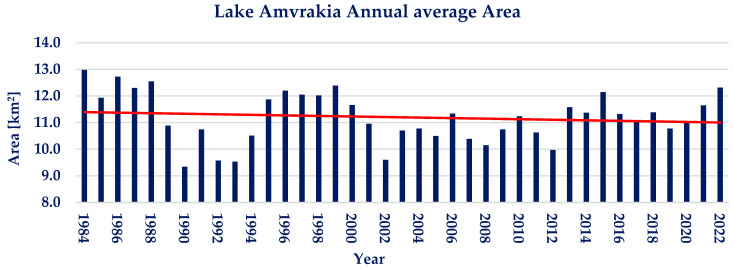
Annual water area average and linear trendline of lake Amvrakia (red line; linear trendline).

The annual average of water area for lake Amvrakia is presented in Figure 9. It is calculated that no significant trend is observed, despite a graphically observed slight negative trend. Lake Amvrakia shows moderate interannual water area variations during the examined 39 years, with the annual water area varying between −16.62% and +13.69% from the annual water area average of 11.2 km^2^. The Pettitt test conducted on the available annual timeseries does not show a statistically significant point of change. Moreover, the Mann–Kendall test resulted in *p* ≈ 1 and Ζ_ΜΚ_ = −0.73, showing a nonsignificant negative trend.

#### 3.1.3. Seasonality Trend Analysis

The investigation of water area seasonality in lake Trichonis, during the available timeseries of 39 years, shows that there is no significant variation between wet and dry periods. More explicitly, the average water area of the lake for the wet periods is 92.17 km^2^, while for the dry ones is 92.16 km^2^. Furthermore, following the results of the seasonality trend analysis, no significant trends or points of change are identified for either period. The wet periods show a *p* ≈ 1 and Z_SK_ = +1.06, depicting a statistically insignificant positive trend, while the dry periods show a *p* ≈ 1 and Z_SK_ = +1.45, depicting again a statistically insignificant positive trend. 

Lake Lysimacheia, during the examined timeseries, shows a slight variation between wet and dry periods. The average wet period water area is calculated at 10.84 km^2^, while the dry periods show an average water area of 10.07 km^2^. Furthermore, following the results of the seasonality trend analysis, no significant trends or points of change are identified for either period. The wet periods show a *p* ≈ 1 and Z_SK_ = −1.46, depicting a statistically insignificant negative trend, while the dry periods show a *p* ≈ 1 and Z_SK_ = −2.85, depicting a statistically insignificant negative trend.

Lake Ozeros, during the examined timeseries, shows a slight variation between wet and dry periods. The average wet period water area is calculated at 9.06 km^2^, while the dry periods show an average water area of 8.97 km^2^. Furthermore, following the results of the seasonality trend analysis, no significant trends or points of change are identified for either period. The wet periods show a *p* ≈ 1 and Z_SK_ = −1.72, depicting a statistically insignificant negative trend, while the dry periods show a *p* ≈ 1 and Z_SK_ = +0.33, depicting a statistically insignificant positive trend. 

Lake Amvrakia, during the examined timeseries, shows a slight variation between wet and dry periods. The average wet period water area is calculated at 11.19 km^2^, while the dry periods show an average water area of 11.05 km^2^. Furthermore, following the results of the seasonality trend analysis, no significant trends or points of change are identified for either period. The wet periods show a *p* ≈ 1 and Z_SK_ = −0.04, depicting a statistically insignificant negative trend, while the dry periods show a *p* ≈ 1 and Z_SK_ = −0.48, depicting a statistically insignificant negative trend.

### 3.2. Further Investigation

#### 3.2.1. Precipitation

Lake Trichonis’ basin’s annual precipitation throughout the available examined years, 1984–2016, is found to be on average 1009 mm. The annual precipitation presents a large variability, showing a maximum value of 1633 mm and a minimum of 595 mm, with a standard deviation of 262 mm. The annual precipitation of lake Trichonis’ basin presents none significant points of change or trends, as the Pettitt and Mann–Kendal tests resulted in *p* ≈ 1 and Z_MK_ = 2.80. Seasonal precipitation trends were also tested and no statistically significant trend was found in either the wet or dry periods. Moreover, the Pearson correlation between the annual precipitation and lake area is calculated at 0.05, implying a negligible relationship between the abovementioned variables.

Lake Lysimacheia’s basin’s annual precipitation throughout the available examined years, 1984–2016, is found to be on average 954 mm. The annual precipitation presents a large variability, showing a maximum value of 1325 mm and a minimum of 606 mm, with a standard deviation of 205 mm. The annual precipitation of lake Lysimacheia’s basin presents no significant points of change or trends, as the Pettitt and Mann–Kendal tests resulted in *p* ≈ 1 and Z_MK_ = 2.37. Seasonal precipitation trends were also tested and no statistically significant trend was found in either the wet or dry periods. Moreover, the Pearson correlation between the annual precipitation and lake area is calculated at −0.03, implying a negligible relationship between the abovementioned variables.

Lake Ozeros’ basin’s annual precipitation throughout the available examined years, 1984–2018, is found to be on average 1028 mm. The annual precipitation presents a large variability, showing a maximum value of 1400 mm and a minimum of 632 mm, with a standard deviation of 247 mm. The annual precipitation of lake Ozeros’ basin presents no significant points of change or trends, as the Pettitt and Mann–Kendal tests resulted in *p* ≈ 1 and Z_MK_ = 3.13. The seasonal precipitation trends have also been tested and no statistically significant trend was found in either the wet or dry periods. Moreover, the Pearson correlation between the annual precipitation and lake area is calculated at −0.05, implying a negligible relationship between the abovementioned variables.

Lake Amvrakia’s basin’s annual precipitation throughout the available examined years, 1984–2018, is found to be on average 963 mm. The annual precipitation presents a large variability, showing a maximum value of 1410 mm and a minimum of 587 mm, with a standard deviation of 198 mm. The annual precipitation of lake Amvrakia’s basin presents no significant points of change or trends, as the Pettitt and Mann–Kendal tests resulted in *p* ≈ 1 and Z_MK_ = 1.60. The seasonal precipitation trends have also been tested and no statistically significant trend was found in either the wet or dry periods. Moreover, the Pearson correlation between the annual precipitation and lake area is calculated at 0.05, implying a negligible relationship between the abovementioned variables.

**Figure 10:**
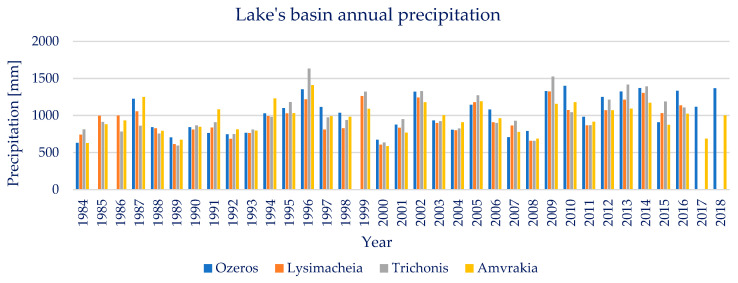
Annual precipitation of the four lakes’ basins.

Overall, regarding the four lakes’ basins’ annual and seasonal precipitation, there is no significant change detected, as far as points of change and trends are concerned. Moreover, the available timeseries of precipitation and the remotely sensed lake water area, do not show a significant value of Pearson correlation between them. The annual precipitation timeseries of the four basins are presented below, in Figure 10.

#### 3.2.2. Temperature

Lake Trichonis’ basin’s annual average temperature throughout the available examined years, 1984–2021, is found to be on average 15.36 °C. The annual average temperature presents a moderate variability, showing a maximum value of 16.49 °C and a minimum of 14.37 °C, with a standard deviation of 0.58 °C. The annual average temperature of lake Trichonis’ basin presents no significant points of change or trends, as the Pettitt and Mann–Kendal tests resulted in *p* ≈ 1 and Z_MK_ = 4.65. The seasonal temperature trends have also been tested and no statistically significant trend was found in either the wet or dry periods. Moreover, the Pearson correlation between the annual average temperature and lake area is calculated at 0.42, implying a weak relationship between the abovementioned variables.

Lake Lysimacheia’s basin’s annual average temperature throughout the available examined years, 1984–2021, is found to be on average 15.36 °C. The average temperature presents a moderate variability, showing a maximum value of 16.49 °C and a minimum of 14.37 °C, with a standard deviation of 0.58 °C. The annual average temperature of lake Lysimacheia’s basin presents no significant points of change or trends, as the Pettitt and Mann–Kendal tests resulted in *p* ≈ 1 and Z_MK_ = 4.52. The seasonal temperature trends have also been tested and no statistically significant trend was found in either the wet or dry periods. Moreover, the Pearson correlation between the annual average temperature and lake area is calculated at −0.22, implying a weak inverse relationship between the abovementioned variables.

Lake Ozeros’ basin’s annual average temperature throughout the available examined years, 1984–2021, is found to be on average 15.36 °C. The annual average temperature presents a large variability, showing a maximum value of 16.49 °C and a minimum of 14.37 °C, with a standard deviation of 0.58 °C. The annual average temperature of lake Ozeros’ basin presents no significant points of change or trends, as the Pettitt and Mann–Kendal tests resulted in *p* ≈ 1 and Z_MK_ = 4.45. The seasonal temperature trends have also been tested and no statistically significant trend was found in either the wet or dry periods. Moreover, the Pearson correlation between the annual average temperature and lake area is calculated at 0.07, implying a negligible relationship between the abovementioned variables.

Lake Amvrakia’s basin’s annual average temperature throughout the available examined years, 1984–2021, is found to be on average 14.69 °C. The annual average temperature presents a large variability, showing a maximum value of 16.88 °C and a minimum of 14.69 °C, with a standard deviation of 0.58 °C. The annual average temperature of lake Amvrakia’s basin presents no significant points of change or trends, as the Pettitt and Mann–Kendal tests resulted in *p* ≈ 1 and Z_MK_ = 4.50. The seasonal temperature trends have also been tested and no statistically significant trend was found in either the wet or dry periods. Moreover, the Pearson correlation between the annual average temperature and lake area is calculated at 0.01, implying a negligible relationship between the abovementioned variables.

**Figure 11:**
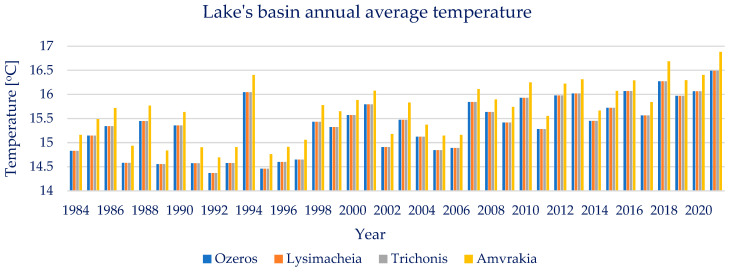
Annual average temperature of the four lakes’ basins.

Overall, regarding the four lakes’ basins’ annual average temperatures, there is no significant change detected, as far as points of change and trends are concerned. Moreover, the available timeseries of temperature and the remotely sensed lake water area, do not show a significant value of Pearson correlation between them. The annual average temperature timeseries of the four basins are presented below, in Figure 11. 

Since temperature is considered the climate variable mostly connected to climate change, there was an additional test conducted. The entire ERA-5 Land monthly average temperature timeseries are tested for significant points of change and trends detection. Yet, none are found, at the 95% or 90% confidence interval, as the Pettitt and Mann–Kendal test concluded in *p* = (0.29, 0.28, 0.34, 0.40) and Z_MK_ = (1.49, 1.49, 1.40, 1.36) for the basins of Trichonis, Lysimacheia, Ozeros, and Amvrakia, respectively.

#### 3.2.3. Land Cover

The examined land cover datasets, describing years 1990 and 2018, show a generally stable land use pattern across the four lakes’ basins. Specifically, human environment land uses, such as motorways and urban areas, gain small portions, from 1% to 10% of each basin’s total area. Additionally, natural vegetation, such as grasslands and forests, also gain small portions, accounting for 1% to 3% of each basin’s total area. In all four lakes’ basins, cultivated areas seem to decline, favoring the abovementioned human environment and natural vegetation uses. The reduction of cultivated areas ranges from 2% to 12% across the four lakes’ basins. As no significant change is detected in any lake basin, it is evident that there will be negligible or small differentiations of the water use regimes across the four basins. This is confirmed by the water area analysis conducted in Section 3.1, where no significant change or trend patterns were detected.

## 4. Discussion

Before discussing the key findings of this research, a few constraining factors should be remarked on as the study limitations. As described in Section 3.1, the four lakes’ water area are found to have been relatively stable. Only small fluctuations are recorded, ranging from −16% to +16% in lake Amvrakia, and from −0.5% to +0.3% in lake Trichonis. A sensitivity analysis was conducted by the authors, but its results are explicitly limited by the small water area change range. All four lakes showed near zero sensitivity [−5%, +5%] to precipitation and temperature, when examined annually. Even when water area sensitivity is examined for larger temporal periods, no specific pattern can be identified. For instance, when examined for the 5-year average water area sensitivity to precipitation and temperature, the results are incoherent. Consequently, this research argues that the small range of change recorded during the period 1984–2022, poses a limit to presenting a well-established sensitivity analysis. Additionally, there is a lack of tools to examine the lakes relationships and interconnections with groundwater, which may also contribute to the observed water content stability. Another aspect which should be addressed in future research is the evaluation of the underlying uncertainties of the data used in the present study. However, since all data sources have been extensively evaluated and used in different case studies and scopes, the underlying uncertainty is judged to be minor.

The present research attempted to identify and evaluate potential quantitative changes in the water content of four natural lakes of western Greece and links to climate variation. Furthermore, the investigation assessed climate variables, such as precipitation and temperature, perceived as highly connected to climate change. Finally, land cover and land use changes were also assessed, since they have strong interconnections with water resources. The overall results are concisely presented in Table 2 and discussed below.

Neither the water area of the four lakes, nor the climatic variables of their basins assessed—precipitation and temperature—show statistically significant annual or seasonal trends or breakpoints during the examined timeseries from 1984 to 2016–2022. The findings of the present research are in agreement with a long-term drought trend analysis over Greece, where no statistically significant trend was identified [20]. Moreover, land cover has remained relatively stable between 1990 and 2018 for the four lake basins, with only small increases in artificial and natural vegetation areas and decreases of cultivated ones. Taking also into account the seasonal stability of the water area timeseries, it is argued that the hydrologic regime is stable within the lake basins. 

Similar research, conducted in different case studies, has associated lake water level decrease with an intensification of agricultural land and water usage [14,15], and not with climatic variables, when the latter remain relatively stable. Such are the present cases of the four lake basins of western Greece, where no significant change of climatic variables is observed. Additionally, the fact that land cover, and especially agricultural land, has remained stable are converging to the long-term stability of the lake water areas, and thus water contents. The nearest case study examined by similar research was lake Prespa, a transboundary natural lake, located in the borders of Greece, Albania, and Northern Macedonia [38]. Although lake Prespa is subject to a century-long decrease in water content, it is suggested that increased irrigation of agricultural land has significantly contributed to a more intense rate of water storage loss [38]. Moreover, another study, examining arid lakes in China, has indicated human uses, and alterations of the basin’s hydrology, as major drivers of lake water loss [69].

According to the 6th IPCC report, the majority of Mediterranean basins are projected to have reduced discharges, while lake water levels are also expected to decline [11]. Moreover, the IPCC report argues for significant (at 0.1 level) trends regarding annual precipitation (decreasing) and annual average temperature (increasing) over western Greece [10]. The abovementioned findings could not be verified by the present research, either regarding annual precipitation, which was based on in-situ data, or the annual temperature of the basins, which was calculated from the ERA5-Land reanalysis data. Therefore, an interesting finding emerges, that different datasets and statistical approaches over the same variables, may lead to divergent results. The present research’s attempt to capture potential quantitative changes of lake water exploited the finest resolution of open access data, 30 m Landsat imagery for lake water area and a dense network of rain gauge stations. Taking into account, the divergent results of this case specific study and of global or regional datasets, the need for extensive validation of the latter is stressed. Furthermore, the need for systematic and efficient downscaling and upscaling approaches is evident, in order to close the gap between local, regional, and global monitoring, and forecasting of hydroclimatic variables.

## 5. Conclusions

The main findings of this research are concentrated below:

- Between 1984 and 2022, the maximum and minimum water area variations compared to average water area for lakes Trichonis, Lysimacheia, Ozeros and Amvrakia, were +1.38% to −3.86%, +39.61% to −32.37%, +4.81% to 4.43% and +19.34% to −19.71% respectively.
- Annual average trend analysis conducted on the Landsat derived water area timeseries, resulted in *p* ≈ 1 and Z_MK_ equal to −0.33, −5.07, +0.36 and +1.87 for the four lakes respectively, depicting the absence of statistically significant quantitative trends.
- Seasonal trend analysis conducted on the Landsat derived water area timeseries did not again show a statistically significant quantitative trend.
- The climate variable of precipitation did not reveal a statistically significant trend during years from 1984 to 2016–2019, in annual and seasonal scale.
- The climate variable of temperature did not reveal a statistically significant trend during years from 1984 to 2022, in annual, seasonal and monthly scale.
- Land use change from 1990 to 2018 revealed a generally stable land environment, with neglible to small increases of artificial and natural areas with a simultaneous decrease of agricultural areas.

The abovementioned points align towards the observed stability of the water area, which was used as an indicator of the lake water storage. Additionally, the stability of the climatic variables of precipitation and temperature was depicted with the use of statistics. Furthermore, the land use changes were assessed and found mostly stable in the catchments of the four lakes examined. These findings strengthen recent similar research, which examined catchments with intense land use alternations and relatively stable climatic variables, resulting in significant lake water storage change. Thus, it is argued that land uses and human interventions to the water cycle are a major driver of pressures to water resources. Regarding the hydrologic implications of the present research, the results depict a stable natural and human environment in the examined catchments. However, the constant monitoring of the associated variables is necessary in order to ensure early and solid identification of change and breakpoints.

The present research successfully demonstrates the combination of different Earth Observation tools and practices, towards the monitoring of regional hydrologic regimens. More specifically, this research makes ground to novel remote sensing and hydrological approaches in Greece, a climatically diverse Mediterranean country. It demonstrates an efficient practice of merging earth observation and in-situ data to create consistent and long timeseries of hydrologic and climatic variables. The exclusive use of open access data promotes the applicability of the proposed approach. The capabilities of open access data and water resources monitoring are expected to be more vital for the years to come, as water demand and climatic pressures are expected to intensify, even if their impacts are not yet traceable in several regions. The use of statistical tools is considered important, in order to trace significant alternations of water regimes and correctly track, each specific case’s causes. Moreover, the examined Earth Observation data sources, when validated with ground truth data, may be significant inputs for successful climate and hydrological forecasting.

## Figures and Tables

**Table 1:** Landsat 4, 5, 7, 8, and 9 missions’ Green and SWIR bands used.

Landsat	Green [μm]	SWIR [μm]
4, 5—Thematic Mapper	B2 (0.52–0.60)	B5 (1.55–1.75)
7—ETM+	B2 (0.52–0.60)	B5 (1.55–1.75)
8, 9—OLI	B3 (0.53–0.59)	B6 (1.57–1.65)

**Table 2:** Overall statistical analysis results; annual timeseries.

Lake/Basin	Water Area		Precipitation		Temperature	
	*p*	Z_MK_	*p*	Z_MK_	*p*	Z_MK_
Trichonis	≈1	1.84	≈1	2.80	≈1	4.65
Lysimacheia	≈1	−2.06	≈1	2.37	≈1	4.52
Ozeros	≈1	−0.60	≈1	3.13	≈1	4.45
Amvrakia	≈1	−0.73	≈1	1.60	≈1	4.50

## Data Availability

All freely available data are mentioned in section on Data and Methods.
